# Identification of the Novel *Streptococcus equi* subsp. *zooepidemicus* Sequence Type 525 in Donkeys of Abruzzo Region, Italy

**DOI:** 10.3390/pathogens12060750

**Published:** 2023-05-23

**Authors:** Maria Chiara Cantelmi, Carmine Merola, Daniela Averaimo, Alexandra Chiaverini, Francesca Cito, Antonio Cocco, Giovanni Di Teodoro, Maria Elisabetta De Angelis, Daniela Di Bernardo, Davide Auzino, Antonio Petrini

**Affiliations:** 1 Istituto Zooprofilattico Sperimentale dell’Abruzzo e Molise “G. Caporale”, Campo Boario, 64100 Teramo, Italy; m.cantelmi@izs.it (M.C.C.); d.averaimo@izs.it (D.A.); a.chiaverini@izs.it (A.C.); f.cito@izs.it (F.C.); a.cocco@izs.it (A.C.); g.diteodoro@izs.it (G.D.T.); m.deangelis@izs.it (M.E.D.A.); a.petrini@izs.it (A.P.); 2 Department of Bioscience and Technology for Food, Agriculture and Environment, University of Teramo, 64100 Teramo, Italy; 3 Department of Veterinary Medicine, University of Teramo, 64100 Teramo, Italy; 4 Freelance Veterinary Practitioner, 65019 Pescara, Italy; iodaniela@hotmail.it (D.D.B.); davideauzino@gmail.com (D.A.)

**Keywords:** emerging pathogens, genomic sequencing, bacterial infections

## Abstract

*Streptococcus equi* sub. *zooepidemicus* (SEZ) is described as a commensal bacterium of several animal species, including humans. Growing evidence supports the potential role of SEZ in the onset and progression of severe clinical manifestations of diseases in horses and other animals. In the present communication, we describe the diagnostic procedure applied to characterize the streptococcal infections caused by a novel SEZ sequence type (ST525) in donkeys raised on a farm in Abruzzo, Italy. The diagnostic process began with anamnesis and anatomopathological analysis, which revealed a severe bacterial suppurative bronchopneumonia associated with systemic vascular damage and haemorrhages. Then, SEZ infection was confirmed by applying an integrative diagnostic strategy that included standard bacterial isolation techniques, analytical tools for bacteria identification (MALDI-TOF MS), and molecular analysis (*q*PCR). Furthermore, the application of the whole-genome sequencing approach helped us to identify the bacterial strains and the virulence factors involved in animal diseases. The novel SEZ-ST525 was identified in two cases of the disease. This new sequence type was isolated from the lung, liver, and spleen in Case 1, and from retropharyngeal lymph nodes in Case 2. Moreover, the presence of the virulence gene *mf2*, a virulence factor carried by prophages in *Streptococcus pyogenes*, was also found for the first time in an SEZ strain. The results of the present study highlight the need to apply an integrated diagnostic approach for the identification and tracking of pathogenic strains of SEZ, shedding new light on the re-evaluation of these bacteria as a causative agent of disease in animals and humans.

## 1. Introduction

*Streptococcus equi* subsp. *zooepidemicus* (SEZ) is a Lancefield group C β-haemolytic *Streptococcus* that is usually considered a commensal bacterium of the oral cavity, pharynx, and respiratory tract of horses [1]. This bacterium can act as an opportunistic pathogen of the respiratory and reproductive tract of horses following, for example, virus infection, heat stress, or tissue injury, causing diseases ranging from mild-to-severe pneumonia, pleuropneumonia, strangles-like diseases, and endometritis [2]. SEZ shares 98% DNA homology with *Streptococcus equi* subsp. *equi* (SEE), but can be differentiated microbiologically by its ability to ferment lactose and sorbitol but not trehalose [3]. However, these biochemical tests can be time-consuming and cause undue delay in obtaining a confirmatory diagnosis of bacterial diseases [4]. For this reason, an analytical tool such as matrix-assisted laser desorption/ionization time-of-flight mass spectrometry (MALDI-TOF MS) can represent a reliable, rapid, and cost-effective approach for the identification of bacteria, including *Streptococcus equi* subsp. *equi* and SEZ [5]. The population of SEZ strains is highly diversified, and the presence of novel superantigens could influence the severity of illness in infected animals. SEZ is also a pathogen of other mammalian species, including livestock, carnivores, and humans [6,7,8]. In the last few years, several SEZ outbreaks of haemorrhagic pneumonia characterized by a high mortality rate have been described in large dog populations [9]. Moreover, many reports of SEZ bacteraemia, meningitis, and arthritis in people have frequently been associated with direct contact with infected animals such as horses, dogs, and guinea pigs [10,11,12]. Moreover, a severe outbreak of SEZ infection due to the consumption of unpasteurized milk dairy has been recently reported in the Abruzzo region, Italy [13]. A total of 37 clinical cases associated with SEZ infections were reported, and 5 patients died because of severe meningitis [13]. SEZ has also been recently identified as the causative agent of endometritis in donkey breeding farms [14]. However, to the best of our knowledge, there are no reports of severe cases of pneumonia in donkeys caused by SEZ. 

The donkey (*Equus asinus*) population in Italy has significantly increased in the last few years, reaching almost 53,500 animals [15]. The potential reasons beyond its significant growth are related to the re-evaluation of donkey milk as a promising “functional food”, representing a suitable alternative to human milk, especially for infants with cow milk protein allergies [16]. Broadly speaking, donkeys can suffer from a similar range of infectious diseases as horses, and they are often treated as “small horses”; however, there are several differences that must be taken into consideration when analysing the impact of infectious diseases on donkey’s health [17]. First of all, donkeys may have different susceptibilities to certain infectious agents, and they usually have mild clinical manifestations compared to horses [18]. Early identification of the disease in donkeys can offer more challenges than in horses because dullness, depression, and anorexia may be the only clinical signs exhibited. As a result, the animal may be in a severe stage of a disease before it can be noticed by the owners. In the present study, we describe the diagnostic process applied to characterize the streptococcal infections caused by a novel SEZ sequence type (ST525) in donkeys raised on a farm in Abruzzo, Italy. The diagnostic process began with history taking and anatomopathological investigations. Then, the following steps were represented by an integrated methodological approach based on standard bacterial isolation techniques, analytical tools for bacteria identification (MALDI-TOF MS), and molecular techniques (qPCR). Furthermore, the application of the whole-genome sequencing approach helped us to characterize the bacterial strains and the virulence factors involved in animal diseases.

## 2. Anamnesis

A total of four donkeys living on a farm in Abruzzo, Italy died between March and April 2022. This farm also housed 15 pleasure horses on various paddocks with full access to the outdoors both day and night. The horses were strictly separated from the donkeys. The age of the donkeys ranged from 3 to 7 years. None of the animals had been vaccinated for any infectious disease in the last two years. They were dewormed and received professional medical care such as evaluation and treatment of teeth or hooves. The owner reported that donkeys were moved from the paddock to a temporary repair facility due to the sudden drop in environmental temperature approximately one month before the onset of clinical signs. 

The first donkey died about 24 h after showing respiratory signs. Unfortunately, due to the animal’s sudden and unexpected death, no veterinary care was applied and the carcass was not screened for any disease. The second animal, Case 1, developed respiratory clinical signs and died after 24 h. On this animal, necropsy was performed to evaluate gross lesions in order to investigate bacterial and viral diseases, and an antimicrobial susceptibility test was also performed after the isolation of *Streptococcus* spp. 

The third and fourth animals were treated according to the results of the antimicrobial susceptibility test, with a third-generation cephalosporin (ceftiofur) used according to the equine label dosage for streptococcal infections (2.2 mg/kg intramuscularly every 24 h) [19]. However, after a slight improvement in medical condition, both donkeys died approximately 72 h later, and are presented as Case 2 and Case 3 in this study. 

## 3. Clinical Signs, Necropsy, and Histopathology

The affected animals showed severe respiratory signs, including serous nasal discharge, dyspnoea, and severe apathy, as well as coughing and pyrexia. 

In Case 1, postmortem examination revealed pleural effusion with subpleural haemorrhages of the lungs and interstitial pulmonary oedema. No significant gross lesions were observed in other thoracic and abdominal organs. Indeed, in the postmortem examination of Case 2, the lungs were wet and failed to collapse. Pleural and epicardial petechial and ecchymotic haemorrhages were described. A large amount of haemorrhagic foamy fluid was present in the trachea and bronchial airways on cut sections. Petechial haemorrhages were also present on the Glisson’s capsule of the liver and in the renal cortex. The spleen and neuroparenchyma were markedly congested. The retropharyngeal lymph nodes were swollen, oedematous, and dark red, and the palatine tonsils were markedly enlarged. No other significant gross lesions were found in the thoracic and abdominal cavities. In Case 3, the lungs had whitish lardaceous and fleshy nodules of approximately 1–2 cm in diameter, localized with a multifocal distribution on the ventral portion of cranial lobes.

For histology, tissue samples were collected and fixed in 10% neutral-buffered formalin (NBF) and routinely processed. Sections of 2 µm in length from all specimens were cut with a rotative microtome (Leica Biosystems, Richmond, IL, USA), mounted on glass slides (Bio-Optica, Milan, Italy), and then stained with standard haematoxylin and eosin (HE). All sections were scanned under an Aperio CS2-Digital Pathology Scanner (Leica Biosystems, Richmond, IL, USA) and visualized for detection of histopathological lesions. 

**Figure 1:**
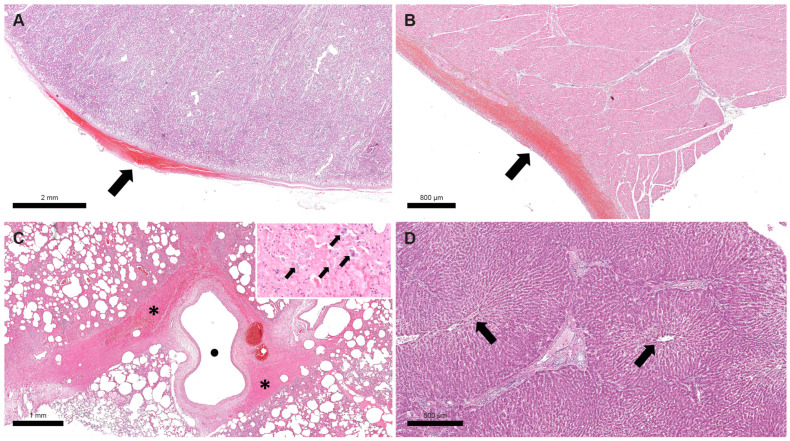
Donkey, Case 1. Haemorrhagic foci were detected in the renal capsule ((**A**), black arrow) and epicardium ((**B**), black arrow). Perivascular haemorrhage was visible in lung along with diffuse hyperaemia and alveolar oedema ((**C**), black asterisks indicate haemorrhagic foci and black dot indicates an empty blood vessel). At higher magnification, many bacteria were visible in the exudate and in lung alveolar macrophages ((**C**), black arrows in the image inset). Degeneration of hepatocytes mainly visible in the central part of lobules, near the central veins ((**D**), black arrows indicate central veins).

Histological lesions of Case 1 and 2 largely overlapped. The main microscopic findings of both donkeys consisted of severe multifocal subcapsular and epicardial haemorrhages in the kidneys and heart, respectively; moderate multifocal to coalescent suppurative bronchopneumonia with multifocal alveolar and interstitial haemorrhages in the lungs; moderate multifocal hepatic degeneration, especially in the central part of the hepatic lobules; and diffuse sinusoid hyperaemia. Numerous slightly basophilic bacteria were evident in airways and in the bronchoalveolar exudates (Figure 1). The lesions described above were consistent with a severe bacterial suppurative bronchopneumonia associated with systemic vascular damage and haemorrhages, suggestive of a septicaemic process. 

Histologic examination of Case 3 showed severe multifocal granulomatous pneumonia with prominent eosinophilic exudate and lung atelectasis. The granulomatous foci consisted of nodules composed of many basophilic fungal hyphae in the central part surrounded by numerous epithelioid macrophages, eosinophils, lymphocytes, rare neutrophils, and occasional multinucleated Langhans-type giant cells. These features were suggestive of multifocal granulomatous mycotic pneumonia. In all three cases, other organs did not show evident microscopic lesions.

## 4. Bacterial Isolation and Identification

Lungs, liver, spleen, retropharyngeal lymph nodes, and guttural pouch samples were obtained aseptically and were used for routine aerobic and microaerophilic bacterial cultures. The samples collected at necropsy were aseptically streaked for isolation onto mannitol salt agar, MacConkey agar, and 5% sheep blood agar plates, incubated at 37 ± 1 °C for 24–72 h. An additional blood agar plate and chocolate agar plate were incubated at 37 ± 1 °C for 24–72 h in microaerophilic conditions using 5–10% CO_2_. Bacteria that grew under aerobic and microaerophilic conditions were identified as *Streptococcus* spp. by morphology, Gram stain, catalase, and oxidase tests. In Case 1, *Streptococcus* spp. was isolated from the lungs, liver, and spleen. In Case 2, *Streptococcus* spp. bacteria were isolated from lungs, retropharyngeal lymph nodes, and guttural pouch samples. In Case 3, *Streptococcus* spp. genes were not isolated in any conditions. 

Then, pure cultures were obtained by transferring colonies into new blood agar plates and incubating at 37 ± 1 °C for 18–24 h. Representative colonies were identified as SEZ using MALDI-TOF MS (MALDI Biotyper^®^, Bruker Daltonics Gmbh & Co. KG, Bremen, Germany). Isolated colonies were frozen and stocked at −80 °C for the following DNA extraction and sequencing. 

## 5. Antimicrobial Susceptibility Test

In Case 1, antibiotic susceptibility was determined by a Kirby–Bauer disk diffusion assay for SEZ, as reported in Table 1. The results were interpreted according to the Clinical and Laboratory Standards Institute (CLSI) guidelines. 

## 6. Differential Diagnosis

Molecular investigations were also carried out to exclude viral infections, including equine viral arteritis, equine herpesviruses, flaviviral encephalitis, and equine influenza. In Case 1, all the analysed specimens were negative for the tested pathogens. In Case 2 and Case 3, the spleen and lungs were positive for equine herpesvirus 1.

## 7. Molecular Investigation

To confirm the diagnosis of SEZ, a qPCR assay was implemented based on an available commercial kit, namely *Streptococcus equi* subspecies *zooepidemicus* genesig^®^ Real-Time PCR assay (PrimerDesign genesig Kit, Southampton, UK). The Real-Time PCR kit targeted against the sorbitol-6-phosphate 2- dehydrogenase (*sorD*) gene was used according to the manufacturer’s instructions. 

In Case 1, SEZ DNA was detected in the lungs and spleen. In Case 2, the qPCR assay identified SEZ DNA in the retropharyngeal lymph nodes, brain, small intestine, tonsils, and guttural pouches. In Case 3, SEZ DNA was not detected in any tested sample.

## 8. DNA Extraction and Sequencing

DNA was extracted according to the method by [20], with minor modifications, using the QIAamp DNA minikit (Qiagen, Hilden, Germany), and quantified using the Qubit double-stranded DNA (dsDNA) high-sensitivity (HS) assay kit (Thermo Fisher Scientific, Waltham, MA, USA).

Next-generation sequencing (NGS) was performed through the Illumina platform (Illumina, San Diego, CA, USA) with a setting of 300 cycles (150 bp paired-end reads) [21]. After reads quality check, species confirmation of the strains was performed by the KmerFinder tool [22]. All strains were confirmed as SEZ. The sequence type (ST), calculated using the MLST scheme (https://pubmlst.org/, accessed on 15 April 2023), resulted in a new ST525. Using ABRicate (https://github.com/tseemann/abricate, accessed on 15 April 2023), the *mf2* gene, which encodes for an deoxyribonuclease phage-associated, was found in three strains, as reported in Table 2; meanwhile, no antimicrobial resistance gene or intact phage were detected using the ResFinder [23] and Phaster tools [24], respectively. In order to confirm the correlation between human SEZ strains, a single-nucleotide polymorphism (SNP) analysis was performed through the CFSAN pipeline, using CP001129 as reference [25]. The clustering analysis revealed that the four strains were close to each other (0–3 SNPs), leading the involvement of a unique source strain in observed cases. One representative genome was deposited in GenBank under the following Bioproject accession number PRJNA946547. 

## 9. Discussion

In the present study, a new SEZ-sequence type, namely ST525, was reported as the causative agent of disease in donkeys. *Streptococcus equi* sub. *zooepidemicus* is ascribed as the most frequently recovered bacterium from the oropharynx of horses [26]. As a consequence of the relatively high prevalence of SEZ in samples taken from healthy horses, this bacterium is often not considered an aetiological agent of disease. However, growing evidence supports the key role of SEZ in the development of severe clinical manifestations of diseases in horses and other animals.

Recently, cases of respiratory disease in working horses of Ethiopia, characterized by coughing, nasal discharge, or altered respiration, were significantly associated with the presence of SEZ, with no evidence for the involvement of viral pathogens [27]. Moreover, the isolation and identification of SEZ strains in other animal species provide an opportunity to examine the potential contribution of this agent in causing disease. For example, an important outbreak, caused by a SEZ clustered with ATCC 35246, inducing high mortality in swine herds, was recently reported in multiple locations in USA and Canada [28]. The SEZ strain ST236 was the dominant strain type recovered from cases of mastitis in goats and sheep in Spain, and none of the caprine and ovine isolates had been previously recovered from horses [29]. In an outbreak of clinical mastitis observed in Italy in dairy goats, caprine isolates of SEZ were distinct from equine isolates [30]. Sequencing of the 16S–23S intergenic spacer region and results from *sodA*-*seeI* multiplex PCR supported the identification of isolates as SEZ. Based on random amplified polymorphic DNA typing and *rpoB* and *sodA* sequencing, caprine isolates were indistinguishable from each other, but distinct from equine isolates, indicating that horses were not the source of the mastitis outbreak in goats [30]. The identification of specific strains of SEZ in animal species different from horses may indicate the possibility that SEZ has evolved as a specific cause of disease in sheep and goats rather than in horses. Moreover, the large outbreak of acute fatal haemorrhagic pneumonia in kennel dogs residing in the United Kingdom between 2000 and 2002 provided further evidence of the ability of specific SEZ subgroups (ST123, SzBHS5) to also infect carnivore hosts [9]. 

In this study, we identified, for the first time, the novel SEZ-ST525 in both cases of disease. This new sequence type was isolated from the lung, liver, and spleen in Case 1, and from retropharyngeal lymph nodes in Case 2. The spread of bacterial infections in several animal organs could justify the pathogenicity and virulence of SEZ strain ST525. This assumption is also supported by the presence of the virulence gene *mf2*, a virulence factor carried by prophages in *Streptococcus pyogenes*. In this bacterium, the *mf2* gene encodes for an exoprotein with DNases activity [31]. The expression of DNases in vivo induces the production of antiDNase antibodies after either pharyngeal or skin infection. However, the role of DNases in the pathogenesis of diseases caused by *S. pyogenes* is not clearly understood. For example, in porcine streptococcal infections sustained by *Streptococcus suis*, the presence of DNases represents an evasion factor to neutrophil extracellular traps, which represents a primitive defence mechanism to entrap and kill pathogens [32]. Moreover, the ability of *S. pyogenes* DNases to act as cytolethal distending toxins (CDTs) was also hypothesized. When CDTs are expressed in host cells, they trigger G2/M cell cycle arrest in mammalian cell lines, leading to the enlarged or distended cells for which these toxins are named [31]. In our cases, for the first time, the *mf2* gene was reported in an SEZ strain, but without the identification of intact phages in bacterial genome. As previously stated, the ability of SEZ to infect a wide variety of hosts and tissues poses significant questions in balancing the need to adapt to diverse environments, potentially through the acquisition of mobile genetic elements. Some SEZ strains, including SzBHS5, Sz35246, and SzH70, contain prophages and/or integrative and conjugative elements (ICEs). However, these SEZ prophages do not appear to carry virulence factors [33]. The relatively low frequency of prophages in SEZ genomes is in contrast with the genome of SEE, which contains ICEs and prophages. In fact, it is hypothesized that these elements have played an important role in the formation of the more virulent clonal SEE from its putative ancestor SEZ, by introducing virulence factors and disrupting functions of genes involved in DNA recombination and repair [34]. Moreover, the similarity of 17% of spacer sequences with prophages of *S. pyogenes*, *S. agalactiae*, or *S. dysgalactiae* provides additional evidence that these bacterial species share a common phage pool, which may permit the cross-species exchange of genetic material [35]. The SEZ strain ST525 does not have antimicrobial resistance genes. This evidence is also confirmed by the antimicrobial susceptibility test executed on Case 1, which showed sensitivity to ceftiofur, as also reported by [36] in SEZ strains isolated in mares with fertility problems. Moreover, the efficacy of the antibiotic therapy with ceftiofur could also justify the lack of SEZ identification in Case 3, which showed complete negativity to all executed tests.

To the best of our knowledge, in these case reports, we also described the first isolation and identification of SEZ as the causative agent of respiratory disease in donkeys. In fact, in this animal species, there is still scarce and fragmentary information on the potential virulence of *Streptococcus* spp. bacteria. The only published cases are related to the outbreaks of strangles associated with SEE, as recently reported in China and Ethiopia, and like other animal species, SEZ is unequivocally considered as commensal, and sometimes an opportunistic pathogen [37,38]. In a recent study aimed at evaluating the nasopharyngeal microbiomes in donkeys shedding SEE in comparison to healthy donkeys, SEZ was hypothesized as the predominant commensal *Streptococcus* spp. bacteria in healthy donkeys, and its relative low abundance in donkeys shedding SEE could lead to microbial dysbiosis, which might predispose them to other airway diseases [39]. Donkeys have been reported to act as asymptomatic chronic carriers of SEZ, with conditions being characterized by guttural pouch infection or intermittent nasal discharge [40]. Moreover, SEZ has been identified in the guttural pouches of systemically ill donkeys with empyema and chondroid, suggesting that in certain circumstances, SEZ can act as a primary pathogen in donkeys [41]. Usually, donkeys are susceptible to a similar range of bacterial and viral respiratory diseases as horses. However, their clinical presentation may vary owing to several factors, including their ability to show only nonspecific signs of disease, such as dullness and anorexia [42]. This factor, associated with their nonathletic lifestyle, can mean that subtle changes in the early stages of disease progression may go unnoticed and the pathological conditions are only recognized relatively late and at a severe stage, resulting in a poorer prognosis [43]. In fact, also in this present case, the infected donkeys showed severe and acute respiratory signs only few hours/days before death, and the owner did not notice any clinical differences until the situation was already irreversible. It is also important to note that certain respiratory diseases present differently in donkeys than in horses. The presence of predisposing factors could be also involved in the development of respiratory disease caused by SEZ in different animal species. The transport stress in horses, as well as the transfer to an unfamiliar environment in dogs, are cofactors for the onset of SEZ infection and diseases [7]. In a recent case report of asinine endometritis due to SEZ infections, the authors hypothesized that the improper operations during artificial insemination caused a transient inflammatory response or injury in the lower reproductive tract, which can promote the occurrence of bacterial infection [14]. In donkeys, the gathering and relocation of the animals to new facilities have been particularly associated with increased stress [44,45]. In the present case, donkeys were moved from the paddock to a temporary repair facility, without the presence of other animal species, due to the sudden drops in environmental temperature. According to the information on anamnesis, we can hypothesize that the relocation, as well as the cold, may have acted as predisposing factors to SEZ infection in the donkey population studied. The tolerance of donkeys to cold conditions has often been mistakenly considered high, but these animals are more vulnerable to cold than horses and mules, and temperatures below 57.2 °F (14 °C) should be considered cold for these animals [46]. This condition is potentially related to the fact that the coat of donkeys, unlike horses and mules, does not significantly differ between seasons, with a low capacity for regulation and adaptation to climate changes [46]. Viral infections have also been shown to facilitate bacterial adhesion to respiratory cells [47] and in horses, the presence of the equine influenza virus has been considered a predisposing factor for SEZ infection of the respiratory tract [48]. In donkeys, coinfection of some SEZ strains and asinine herpesvirus 5 (AHV-5) with *Dictyocaulus arnfieldi* has been hypothesized to contribute to clinical disease, but without empirical or experimental evidence [45]. In our case, two dead animals (Case 2 and Case 3) were also positive for equine herpesvirus 1 and one (Case 3) had a potential mycotic infection. However, establishing if the viral infection had triggered the bacterial proliferation, as well as the exact relationship between transfer and cold stress, bacterial and mycotic infection, and virus reactivation, is a difficult task. 

## 10. Conclusions

In the present study, we identified a new sequence type of SEZ strain, namely ST525, involved in respiratory infections of an Italian donkey farm. Moreover, the presence of the bacterial virulence gene *mf2* was also confirmed for the first time in an SEZ strain. This virulence gene, which had a phage origin, is usually related to *Streptococcus pyogenes*, and its occurrence in SEZ-ST525 could justify its high pathogenicity and virulence. This report opened a new and interesting scenario in the study of SEZ strains, which are often just considered commensal and opportunistic pathogens. In fact, the genome diversity of SEZ cannot be captured by traditional phenotypic strain typing methods, which were only able to differentiate SEZ from SEE. This limitation has led to the assumption that all SEZ strains were commensal organisms, even though some strains can be highly virulent, leading to severe disease and even death. The availability of new diagnostic tools, such as genomic sequencing technologies, for the identification and tracking of pathogenic strains of SEZ, can help obtain more accurate data on this neglected pathogen and to find the emergence of new sequence types.

## Figures and Tables

**Table 1:** Antimicrobial susceptibility test of Case 1. The response of stains against different antibiotics was characterized as sensitive (S), intermediate (I), and resistant (R).

Antibiotic	Results
Ceftiofur	S
Tylosin	S
Rifampicin	S
Ampicillin	S
Lincomycin	S
Rifamixin	I
Spiramycin	I
Penicillin	I
Gentamicin	I
Neomycin	R
Amikacin	R
Sulfamethoxazole and Trimethoprim	R
Amoxicillin	R
Tetracycline	R
Streptomycin	R
Aminosidine	R

**Table 2:** Metadata and microbiological characterization of 4 SEZ isolates typed by whole-genome sequencing (WGS).

Case n°	Isolate ID	Matrix	Virulence-Associated Genes	PHASTER	ResFinder
1	2022.TE.33840.1.2	Lung	-	-	-
1	2022.TE.33841.1.2	Liver	*mf2*	-	-
1	2022.TE.33842.1.2	Spleen	*mf2*	-	-
2	2023.TE.1927.1.2	Retropharyngeal lymph nodes	*mf2*	-	-

## Data Availability

All the data generated are described in the present article.
